# Gender differences in tibial microvascular flow responses to head down tilt and lower body negative pressure

**DOI:** 10.14814/phy2.13143

**Published:** 2017-02-27

**Authors:** Jamila H. Siamwala, Brandon R. Macias, Paul C. Lee, Alan R. Hargens

**Affiliations:** ^1^ Department of Orthopedic SurgeryUniversity of CaliforniaSan DiegoCalifornia

**Keywords:** Bone microvascular flow, head down tilt, limb girth, lower body negative pressure, oxygenation, photoplethysmography, spaceflight

## Abstract

The purpose of the investigation was to study lower body negative pressure recovery in response to head down tilt position in men and women. The study examined the primary hypothesis that tibial bone microvascular flow responses to HDT and lower body negative pressure (LBNP) differ in women and men. Nine women and nine men between 20 to 30 years of age participated in the study. Tibial microvascular flow, head and tibial oxygenation and calf circumference were measured using photoplethysmography (PPG), near‐infrared spectroscopy (NIRS) and strain gauge plethysmography (SGP), respectively, during sitting (control baseline), supine, 15° HDT, and 15° HDT with 25 mmHg LBNP. Tibial microvascular flow with HDT increased by 57% from supine position (from 1.4V ± 0.7 to 2.2V ± 1.0 HDT; ANOVA *P* < 0.05) in men but there is no significant difference between supine and HDT in women. Ten minutes of LBNP during 15^o^ HDT restored tibial bone microvascular flows to supine levels, (from 2.2V±1.0 HDT to 1.1V ± 0.7 supine; ANOVA *P* < 0.05) in men but not in women. These data support the concept that there are gender specific microvascular responses to a fluid‐shift countermeasure such as LBNP. Thus, gender differences should be considered while developing future countermeasure strategies to headward fluid shifts in microgravity.

## Introduction

Bone loss during long‐term space missions is a major concern for fracture risk (LeBlanc et al. 2000). The probabilities of osteoporotic fractures due to bone loss is 3.5–31% higher in women compared to 2.8–15% in men (Kanis et al. 2008). These probabilities can be exacerbated in space as longer space missions lasting from 75 to 184 days leading to approximately 20% bone loss in returning space crews (Stupakov et al. 1984; LeBlanc et al. 2000). Long‐term Mars mission consisting of 2–3 years may expose astronauts to 50% loss of bone mineral density particularly in the lower extremities (Holick 2000). Women are more susceptible to bone loss and fractures compared to men as they start with a lower peak bone mass and reach osteoporotic stage faster than men (Vogt et al. 1997). Recent countermeasures such as artificial gravity measures designed to counteract microgravity effects are ineffective and may not take the gender into consideration (Ploutz‐Snyder et al. 2014) .

Recent reviews on spaceflight hemodynamics suggest gender specific cardiovascular adaptations to microgravity environments (Fu et al. 2004; Harm et al. 2001). Women astronauts experience greater losses of plasma volume during spaceflight and greater post‐flight orthostatic intolerance compared to male astronauts (Harm et al. 2001). Moreover, men respond to orthostatic stress with an increase in vascular resistance, while women respond with an increase in heart rate. Therefore it may be necessary to test the effectiveness of countermeasures in both the genders considering the differences in physiology and cardiovascular regulation.

The weight bearing regions such as the hip and tibia show maximum bone loss which may be due to altered bone perfusion due to headward fluid shifts (Hargens and Richardson 2009). Evidence for reduced fracture healing and bone loss is based on histomorphometric analysis of tibial cancellous bone of rats flown on Cosmos flight and parallel ground based hindlimb suspension studies. (Vico et al. 1993, 1995; Jee et al. 1983). It is documented that elderly women with reduced perfusion have increased femoral neck bone loss (Griffith et al. 2011). Therefore altered bone perfusion in the weight bearing regions maybe the main mechanisms of bone loss observed in returning crew. The mechanisms of altered perfusion and bone loss in space in not known. Normally in an upright posture, gravity's effects on columns of fluid causes mean arterial pressure to be approximately 200 mmHg at the feet compared to 100 mmHg at the heart and 70 mmHg at the head (Hargens and Richardson 2009). Lack of gravity in space causes loss of hydrostatic blood pressure gradients from the head to foot, thus equilibrating blood pressures throughout the body and probably affecting blood perfusion of lower extremities (Watenpaugh and Hargens 2011). However, fluid shifts effects on bone perfusion in weight bearing regions of the body do not take gender into consideration.

Head‐down tilt (HDT) is an effective analog for microgravity's effects on macro‐ and microvasculature, and is frequently used in studies concerning physiological adaptation and countermeasure efficacy (Christ et al. 2001). During HDT, the transmural pressures in the arteries of the leg decrease and the vessels vasodilate resulting in increased blood flow (Bayliss 1902). One potential countermeasure for HDT‐induced alterations in hemodynamics is lower body negative pressure (LBNP). Feet‐supported LBNP provides weight bearing and simulates gravitational blood pressure gradients within the body (Hargens et al. 1991). In our previous study, we evaluated the efficacy of LBNP in restoring bone blood flow during microgravity simulated by HDT (Siamwala et al. 2015). When LBNP is introduced during HDT, transmural pressures in the leg are re‐established qualitatively; and cutaneous microvascular flow and tibial bone microvascular flow return to normal levels (Siamwala et al. 2015). In the current study, we sought to determine whether women and men have equivalent tibial hemodynamic responses to LBNP. We hypothesized that men and women have parallel responses in tibial bone microvascular flow, tibial oxygenation, and calf circumference during HDT and LBNP during HDT. The results of this study may help determine if LBNP is an effective countermeasure to headward fluid shifts for both the genders.

## Materials and Methods

### Participants

Eighteen, non‐smoking, healthy participants, 9 women and 9 men, between the ages of 20–40 years were recruited for this study. The Institutional Research Board of the University of California, San Diego approved this study and the participants gave verbal and written consents prior to participating in the study. On the day of the experiment, the participants were asked to wear loosely fitting clothes to prevent any mechanical compression of the leg.

### Experimental protocol

The experimental protocol was similar to our previous study (Siamwala et al. 2015). Briefly, blood flow, tissue oxygenation and calf circumference were measured in men and women participants in the following positions: (1) sitting, (2) supine, (3) head down tilt (HDT), and (4) HDT with 25 mmHg LBNP (HDT + LBNP). An automated blood pressure cuff (Deluxe Automatic Blood Pressure, Microlife USA Inc., FL) was placed around the participant's left arm over the brachial artery; and blood pressure and heart rate were determined in each position. The photoplethysmography (PPG) probe, used to measure blood flow was placed on the antero‐medial surface of the left tibia of the participants and calibrated according to the skin color to get the maximum signal and minimum noise. The near‐infrared spectroscopy (NIRS) probe, used to measure oxygen saturation was placed on the medial surface of the tibia bone, about 4–6 cm distal to the tibial tuberosity and on the middle, flat part of the head. The strain gauge (SG), used to measure calf circumference was positioned around the right calf below the NIRS probe. All participants were grounded throughout the experiment to reduce background noise. Participants were instructed to keep conversation and movement to a minimum throughout the experiment. All other electronic devices were removed to minimize interference. Signals from PPG, NIRS and Strain Gauge were recorded simultaneously.

### Instrumentation and measurements

#### Tibial blood flow

The bone microvascular flow measurements on the tibia were acquired using a PPG system previously validated by Mateus and Hargens (2012) and Sandberg et al. (2005). The PPG probe consists of three main parts: (1) a green light‐emitting diode (LED), (2) an infrared LED, and (3) a photodetector. The wavelengths of the green and infrared lights are 560 and 800 nm, respectively, which are the isobestic points of oxygenated and deoxygenated hemoglobin. Green light has a shorter penetration depth and can measure the skin blood flow. The infrared, with its greater penetration ability, can measure the tibial bone microvascular flow. The refracted light is then detected by the detector. The PPG was connected to a LabView™ data acquisition system. The pulsatile nature of the PPG waveform corresponds to the rhythmic heartbeat, and the AC component of the PPG signal represents the microvascular blood flow. Depending on the body part where the PPG probe is placed, microvascular blood flow can be measured in skin along with either skeletal muscle or bone. In this experiment the PPG probe was placed on anteromedial surface of the left tibia to measure the bone microvascular flow. To account for inter‐subject variability, the bone microvascular flows were expressed as fold change compared to sitting. The probe was secured in place with tape and covered with a foil bandage to reduce external light interference of the photo detector.

### Head and tibial oxygenation

Regional oxygenation saturation (rSO_2_) was measured using a standard clinical near‐infrared spectroscopy (NIRS) instrument (Somanetics INVOS Oximeter, Model 5100C) (Siamwala et al. 2015). The NIRS was used to simultaneously measure oxygenation changes in the head and tibia. The NIRS is a non‐invasive technology used to measure dynamic gaseous exchange in the microvasculature. Typically, the NIRS provides real time data about venous oxygen reserve as it can detect the venous and arterial blood contribution in the ratio of 3:1. The NIRS probe is similar to the PPG probe in that it contains a near‐infrared LED and a photo detector. The NIRS probe emits near‐infrared light at wavelengths that can be absorbed by the deoxygenated and oxygenated hemoglobin (730 and 810 nm), respectively. The ratio of the two measurements is used to determine the rSO_2_. In this experiment, one NIRS probe was placed on the head and another was placed on the upper medial surface of the left tibia. Fresh, new NIRS probes were used for each subject throughout the protocol.

### Calf circumference

The relative calf circumference was measured with a mercury strain gauge connected to a Hokanson photoplethysmograph device (Watenpaugh et al. 2004), which was connected to the LabView™ data acquisition system. The strain gauge was calibrated to 0 V for the baseline measurement taken during sitting position. All subsequent measurements in other positions were relative to the baseline measurements. The sizes of the strain gauge varied with each subject's calf circumference so that it could be securely wrapped around the middle of the right calf.

### Head down tilt table/lower body negative pressure chamber

A head‐down tilt table combined with a LBNP chamber was used as previously described to tilt the participants head‐down and to apply lower body negative pressure (Macias et al. 2015; Siamwala et al. 2015). Briefly the lower body negative pressure chamber was attached by straps to the lower half of a tilting examination table. Participants were inserted horizontally into the chamber up to a neoprene waist seal with an adjustable strap. The other end of the chamber had an opening where the vacuum tube was inserted. An external vacuum system was used to generate negative pressure in the chamber. The level of negative pressure was monitored using a pressure gauge connected to the chamber. An inclinometer was placed on either side of the bed to measure the tilt angle.

### Data analysis

#### Photoplethysmography recordings

The PPG and strain gauge recordings of tibial blood flow and calf circumference were taken and the peak to peak analysis algorithm of root mean squares averages of was carried out using the LabVIEW program in each position as described in our previous study (Siamwala et al. 2015). To ensure that the infrared light primarily recorded bone microvascular flow, the PPG probe was placed on the medial surface of the tibia where there is no musculature and minimal soft tissue (approx. 2 mm thick) (Mateus and Hargens 2012). The body mass index (BMI) of all the participants was within a range of 23–25 and hence the interference from fat was minimized.

### Signal acquisition and analysis

As described previously, the relative values were recorded in Volts at a sampling frequency of 300 Hz and processed using LabVIEW 7 Express 2003 (National Instruments^®^, Austin, Texas) (Mateus and Hargens 2012). PPG peak‐to‐peak average values for the last minute of each position was exported to an Excel sheet and averages calculated. Similarly the strain gauge data were imported to Excel and the calibrated value calculated by subtracting the values from each position from the baseline. In all the cases, the last minute of measurements for each position was averaged. The NIRS data were given as absolute rSO_2_ values. NIRS data were imported into an INVOS Analytics Tool Program (Covidien, Mansfield, MA), then exported to Excel for post analysis processing and calculating the rSO_2_ averages for each position.

### Statistics

Repeated measures analysis of variance (RMANOVA) was used to analyze the bone microvascular, head oxygenation, tibial oxygenation and calf circumference for all the four positions (sitting, supine, HDT, HDT, and LBNP). If a significant main effect was determined, pair‐wise comparisons were conducted to determine individual differences among all conditions using the SPSS software (SPSS, Chicago, IL). Green house‐Geisser corrections were used to correct violations of the sphericity assumption. The comparison between men and women with position and response to lower body negative pressure was made using one way ANOVA using gender as an independent variable. Two tailed paired student *t* tests with Welch correction was also used as appropriate. For each statistical test, the significance was set at *P* < 0.05.

## Results

### Heart rate and mean arterial pressure differences in men and women

**Figure 1:**
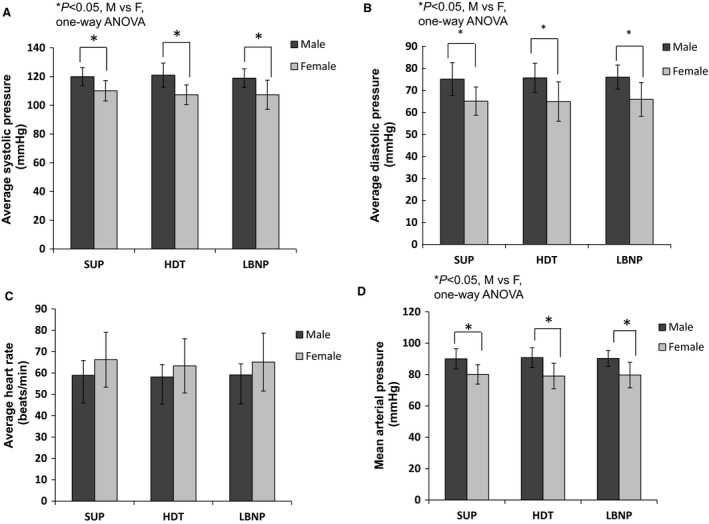
Blood pressure data from the Head down tilt (HDT) and Lower body negative pressure (LBNP) experiment. (A) Systolic measurements in supine, HDT and LBNP position (B) Diastolic measurements in 5 min supine, 5 min HDT and 10 min LBNP position (C) Heart rate measurements in all three positions (D) Mean arterial pressure measurements in all three positions. Data are expressed as mean ± SD in Volts (V). Post hoc comparisons demonstrated that average systolic and diastolic pressure was significantly higher in men than women (*P < 0.05). There was no significant difference in heart rate between men and women determined using unpaired *t* test and one way ANOVA. LBNP, lower body negative pressure.

Average heart rate (HR) and blood pressure (BP) for each position are depicted in Figure 1. The average HR and BP did not change during all test conditions. However there were significant differences between male and women in BP and Mean Arterial Blood Pressure (MABP) determined using one way ANOVA. The supine systolic blood pressure (SBP) and Diastolic blood pressure (DBP) were lower by 10 mmHg in women compared to men. Similarly in the HDT position, the SBP was 14 mmHg lower in women and DBP was 10 mmHg lower than men. The MABP was 12 mmHg and DBP was 10 mmHg lower in women also for the LBNP response. The HR was on an average 5–7 mmHg higher in women for all the conditions than males although this was not statistically significant.

### Bone microvascular flow differences in men and women during LBNP

**Figure 2:**
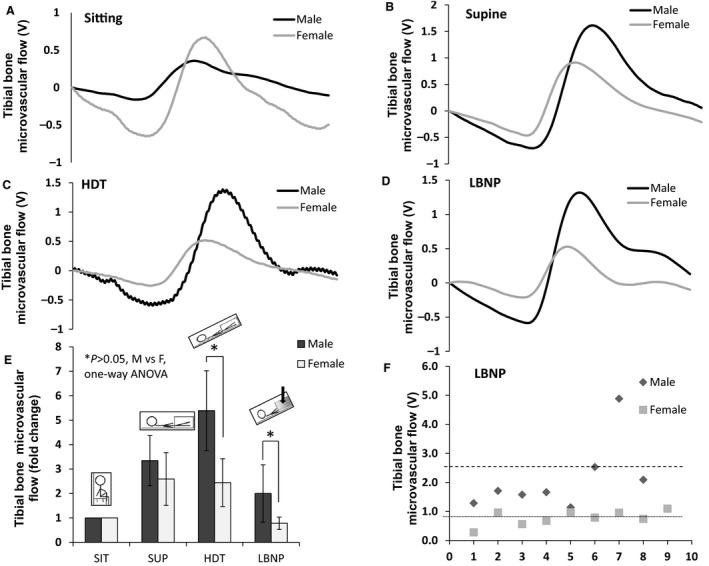
Gender specific responses of tibial bone microvascular flow. (A–D) Comparisons of the male and female averaged waveforms obtained using the Photoplethysmography (PPG) device during the last one min of sitting, supine, HDT, and LBNP in response to tibial bone microvascular flow. (E) Average tibial microvascular flow responses between males and females are represented as mean ± SD in Volts (V). *P < 0.05 for male versus female comparisons. Comparisons between each condition with RMANOVA followed by Bonferroni correction for multiple comparisons. Male and female responses were compared using paired *t* tests with Welch corrections. (F) Scatter plot of all the individual data points of tibial microvascular flow response to lower body negative pressure in male and female participants. LBNP, lower body negative pressure.

The averaged waveforms for tibial bone responses during the last 1 min of sitting, supine, HDT and LBNP of a mean responder men and women are presented in Figure 2. The average tibial bone microvascular flow responses to all four positions and differences between men and women are shown in Figure 2. There was a significant interaction between the conditions and gender as determined by RMANOVA, *P* < 0.001). There was no significant change (*P* > 0.05) in the tibial microvascular flow between different positions going from sitting to supine position or going from supine to HDT in women while in men the tibial microvascular flow increased by 83.3% from supine to HDT (*P* < 0.05, RMANOVA). LBNP restored tibial microvascular flow in male however there was no significant change in tibial microvascular flow in women. Between the men and women, the tibial microvascular flow response to LBNP was significantly different (0.8 ± 0.3V vs. 2 ± 1.2V, *P* = 0.016).

### Head and tibial oxygenation differences in men and women during LBNP

**Figure 3:**
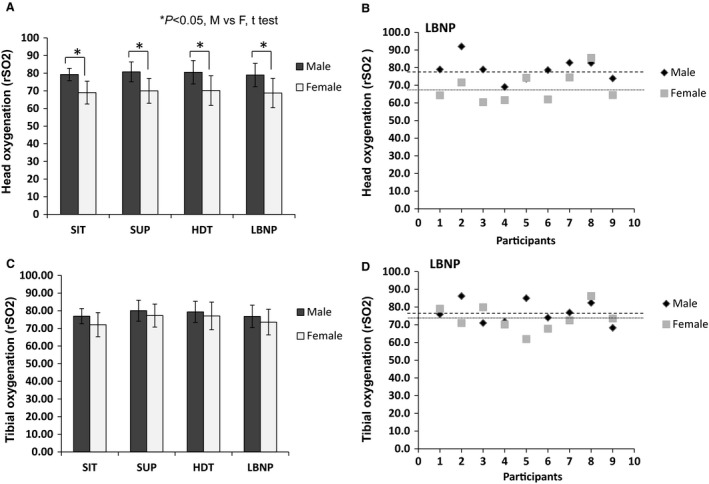
Gender specific head and tibial oxygenation. (A) Head oxygenation (rSO_2_) responses with each condition are presented for both males and females. (B) Individual head oxygenation responses to lower body negative pressure are presented as a scatterplot. (C) Tibial oxygenation (rSO_2_) responses with each condition are presented for both males and females. (D) Individual tibial oxygenation responses to lower body negative pressure are presented as a scatterplot. Male and female responses were compared using unpaired *t* tests with Welch corrections. Data are expressed as means ± SD in Volts.

There was no significant main effect between brain rSO_2_ values in sitting, supine, HDT and HDT + LBNP positions when men and women were combined (Fig. 3). However there were significant differences when pair‐wise comparisons were made between men and women followed by post hoc Bonferroni analyses. Comparisons between men and women responses to LBNP, showed that the rSO_2_ levels in women were significantly reduced compared to men (68.4 ± 8.3 rSO_2_ vs. 78.9 ± 6.6 rSO_2_, *P* = 0.006). The tibial rSO_2_ values for men and women are presented in Figure 3. The men and women comparisons showed that rSO_2_ responses to LBNP were not significant (*P* > 0.05). The rSO_2_ values of women compared to men were (73.5 ± 7.2 rSO_2_ vs. 76.8 ± 6.3 rSO_2_).

### Calf circumference differences in men and women during LBNP

**Figure 4:**
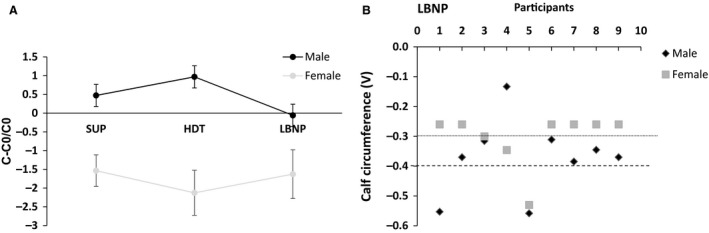
Gender specific calf circumferences. Comparisons between each conditions were made with RMANOVA followed by Bonferroni correction for multiple comparisons. Male and female responses were compared using unpaired *t* tests with Welch corrections. Data are expressed as means ± SD in Volts (V).

Calf circumference measurements using strain gauge plethysmography are shown in Figure 4. As the positions changed from sitting to supine, no significant differences were observed between the different position between the two genders.

## Discussion

Our study demonstrates that there exist gender differences in tibial microvascular flow and oxygenation with HDT and LBNP. Men exhibit a significantly higher tibial microvascular flow response to HDT, and LBNP compared to women. Individual oxygenation responses in the head at all the conditions were similar in both the genders. However, between the genders the oxygenation responses in the head were significantly lower in women compared to men. Calf circumference did not change significantly for both genders in all conditions tested.

We found that gender indeed plays an important role in determining the ability of LBNP to restore tibial microvascular flow to baseline levels. Differences in blood pressure regulation between men and women is well established (Reckelhoff 2001). Blood pressure is 6–10 mmHg higher in men than in pre‐menopausal women of similar ages (Reckelhoff 2001). A recent paper documents significant sex specific differences in cardiovascular regulation during HDT bed rest and artificial gravity. The systolic blood pressure of women is the same after HDT bed rest and artificial gravity while the systolic blood pressure of men dropped to 11 mmHg during artificial gravity (Evans et al. 2015b). Differences in responses to LBNP may also be related to the inherent gender differences in blood pressure regulation. We speculate that apart from the systemic differences in blood pressure regulation, gender differences in the properties of blood vessels may affect the microvascular flow. Gender specific differences in microvascular flow and vasodilatory capacity exist in skin microcirculation in new born preterm infants (24–28 weeks) infants. Male infants have a higher baseline flow compared to female infants (Stark et al. 2008). Similar to our study, where men have higher supine tibial bone microvascular flow, and oxygenation levels compared to women (Figs. 2, 3). The exact mechanism is unclear. In animal studies, gender differences are observed in superoxide concentration and vascular permeability of venules. These variations are associated with gender differences related to gonadal hormones and their receptors (Dantas et al. 2004). Ground‐based microgravity models such as hind limb suspension rat models and HDT show that altered blood pressure leads to changes in local distributions of blood flow and muscle mass (Roer and Dillaman 1990). In humans, the fluid flow within the bone is driven by both blood pressure and mechanical loads resulting in shear stresses that play a major role in signal transduction and mechanical load induced remodeling of bone (McAllister et al. 2000). Vogt et al. (1997) in a classic study show that decreased blood pressure and blood flow in the legs lead to a 3.75% decrease in hip bone mineral density among post‐ menopausal women. Post‐menopausal women experience 1% percent bone loss per year, similar to the 1–2% bone loss per month in astronauts (Keyak et al. 2009).This accelerated bone loss in space may be due to decreased blood flow to the legs with microgravity exposure (McCarthy 2005; Bloomfield et al. 2002). Hence, it is possible that decreased tibial microvascular flow in the legs contributes to bone loss in male crew members.

### Gender difference in cardiovascular responses to head down tilt

Head down tilt (HDT) is used to simulate loss of blood pressure gradient and fluid shifts. In 15^o^ HDT position tibial bone microvascular flows increase significantly in men, but does not change significantly in women. Fifteen degrees HDT is used to induce an acute fluid shift response to study vascular hemodynamics more effectively (Macias et al. 2015). Dimitris and co‐workers recently showed that in men, cardiopulmonary system adapts to −15° HDT positions by increasing systolic, diastolic and mean arterial blood pressure (Galanis et al. 2013). This is further supported by increases in blood pressure and heart rate in rat studies (Musacchia and Fagette 1997). The increase in blood pressure and decrease in heart rate suggest an increase in total peripheral vascular resistance. However, in case of women, Berry and associates show that elderly hypertensive women have a higher peripheral vascular resistance even after adjustment for aortic area. Hence in women, lower blood flow may be due to the incapacity of the microvasculature to increase blood flow when demand increases in the HDT position. In 15^o^ HDT condition in women, it is probable that the intraosseous vessels become less sensitive to circulating catecholamine's due to decreased blood flow as compared to men. Since HDT simulates fluid shifts that occur in microgravity, these results suggest that lower blood pressure in women along with minimal response to fluid shifts and lack of adaptive local vasodilatory capacity may in long term be detrimental to their system, perhaps making them susceptible to fractures and osteoporosis.

### Gender difference in tibial hemodynamic responses to lower body negative pressure

LBNP is currently being evaluated as a potential countermeasure to fluid shifts on the International Space Station. During LBNP about 80% of the negative pressure is transmitted to the bone irrespective of the skin thickness, time and magnitude leading to increase in transmural pressure across the vessel walls, with a concomitant vessel dilation and blood pooling (Olsen and Lanne 1998). The arterial bed is 3% less compliant than the venous bed, hence it is assumed that venous blood is pooled (Rothe 1983). Previous studies suggests differences in blood pressure regulation in men and women (Yang et al. 2012; Evans et al. 2001) following artificial gravity (Evans et al. 2015a). Although the exact mechanisms are currently unknown, the differences in sympathetic and parasympathetic neural firing (Yang et al. 2012), blood pressure regulation (Convertino 1998; Hart et al. 2012), body size and hormones (Evans et al. 2001) may impact the tibial hemodynamic responses to LBNP. The smaller body size in women may also influence the tibial microvascular flow responses to HDT and LBNP. In women, distance from the heart to the feet is shorter than distance from the heart to the feet in taller men. Therefore, capillary blood pressure above the heart level is lower than capillary pressure below the heart level. Because women have reduced small vessel compliance than men (Winer et al. 2001), the microvasculature in the tibia adapts better to the vascular pressures and there are no significant changes in microvascular flow in HDT position. But in case of men, there is a significant increase in tibial microvascular flow due to distance of the leg from the heart and non‐compliant vessels. When LBNP is applied, transmural pressures across the capillary walls increase, resulting in precapillary constriction thus reducing capillary pressure and decreasing in microvascular flow in men. Importantly in women, this effect is more pronounced and the tibial microvascular flow comes back to sitting levels. Gender differences in vascular architecture may also affect transcapillary exchanges. Previous studies have shown that the vascular architecture differs significantly in response to exercise training (Stenger et al. 2012), heart failure, hypertension and diabetes (Juutilainen et al. 2004) in both the genders. For example, in a rat model of hypertension, mean arterial pressure rises in men at an early age and is associated with significant loss of skeletal muscle micro vessels (rarefraction). In women the rise of mean arterial pressure is less and is not associated with rare fraction (Papanek et al. 1998). Exercise training improves capillary density in both the genders, although it increases submaximal performance in men but not in women (Robbins et al. 2009).

### Pre‐menopausal women versus post‐menopausal women's response to lower body negative pressure

LBNP responses may be different in pre‐menopausal and post‐menopausal women. In the current study, all women participants were pre‐menopausal. Premenopausal women have relatively higher vascular stiffness, better left ventricular performance and different autonomic nervous system regulation (Narkiewicz et al. 2005; Hart et al. 2009; Christou et al. 2005). Premenopausal women have lower incidence of cardiovascular disease compared to age‐matched men. However, menopause in women is associated with an increase in cardiovascular disease, suggesting that ovarian hormones play a protective role in the cardiovascular system in premenopausal women. Estrogen improves the arterial wall properties and inhibits development of atherosclerosis by promoting re‐endothelialization, inhibiting smooth muscle cell proliferation, and matrix deposition following vascular injury (Mendelsohn and Karas 2005). Estrogen also reduces systemic vascular resistance, making the vessels more complaint and thereby improves the coronary and peripheral endothelial function (Collins et al. 1995). Interestingly, while supplementation of estrogen improves coronary blood flow in women, estrogen supplementation in men has no effect on coronary blood flow (Leonardo et al. 1997). The women participants in our study were 25 years on average and all were premenopausal.

### Potential underlying mechanisms regulating gender differences in microvascular flow response to LBNP

The response to LBNP occurs through the myogenic responses of the capillaries (Siamwala et al. 2015). Previous studies document a lesser myogenic response in female mice and rat cerebral arteries (Geary et al. 2000a,b), rat muscle arterioles (Huang et al. 1997) and rat coronary arteries (Wellman et al. 1996). The myogenic responses in female mice may be mediated through the estrogen dependent nitric oxide release (Gros et al. 2002). Similarly, in our study, the hemodynamic responses to altered posture and LBNP are less in women compared to men, probably due to differences in myogenic tone and perfusion pressures. The large arterial flow in women is also different compared to men. Recent studies show that the augmentation index in women is twice as high as in men. Because body size in women is smaller compared to men, the shorter distance of wave propagation results in early reflection from peripheral vascular bed and as a consequence early systolic wave return to the heart (Smulyan et al. 2001). Age as a factor that also influences the gender based myogenic responses. A study by Mehta and colleagues shows that age correlates significantly with large artery elasticity (*r* = −0.385, *P* = 0.002) and small artery elasticity (*r* = −0.259, *P* = 0.046) (Mehta et al. 2003). The central pulse pressure and augmentation index are thus related to the body size in women. In short, female vasculature is better adapted to altered hydrostatic pressures and this advantage may be conferred by female body size, vascular architecture and myogenic responses in their vessels.

From the above arguments, it is clear that gender affects the capillary structures and transcapillary exchange. Capillary exchange between the vessels and tissue (J_c)_ is given by Starling‐Landis equation J_c_ = L_p_A [(*P*_c_–*P*_t)_– *σ*_p_ (*π*_c_– *π*_t_)] where J_c_ is the net transcapillary fluid transport, L_p_ is the hydraulic conductivity of the capillary wall, A is the capillary surface area, *P*_c_ is the capillary pressure, *P*_t_ is interstitial fluid pressure, *σ*_p_ is the reflection co‐efficient for protein, *π*_c_ is the capillary blood colloid osmotic pressure and *π*_t_ is the interstitial fluid colloidal osmotic pressure (Hargens and Villavicencio 1995). Up to the age of 65, capillary pressure *P*_c_ is lower in women compared to men. Venous compliance is lower in women compared to men. Reduction in venous compliance results in low transmural pressure in women (Huxley and Wang 2010). Hence at a given pressure, veins in women can accommodate a greater change in blood volume (Lindenberger and Lanne 2007). Thus for a given increase in venous volume, hydrostatic pressures increase more quickly in men compared to women (Shore et al. 1995). The difference in *P*_c_ in men and women is 2.3 mmHg leading to 1 mmHg difference in *π* between men and women. At low pressures, the venous compliance in the lower limbs in men is greater than women, although the capillary filtration co‐efficient (CFC) is higher in women (Lindenberger and Lanne 2007). In a previous study, Lindenberger and Lanne found that reduced LBNP (11, 22 and 44 mmHg) decreases venous compliance in women at low transmural pressures. However at higher transmural pressures, this difference is less evident. Hence the net capillary fluid filtration and CFC is higher in women than in men during LBNP (Lindenberger and Lanne 2007). The addition of LBNP imposes negative pressure to the lower leg and is transmitted to the leg interstitial fluid, although capillary blood pressure remains constant. Negative interstitial fluid pressure with LBNP, combined with constant capillary blood pressure, causes a net Startling force that favors the movement of fluid from the vascular space to the interstitium of the lower leg (Aratow et al. 1993). This explains the decrease in tibial microvascular flow seen during HDT with LBNP.

LBNP normalizes microvascular flow to supine levels in men and sitting levels in women during simulated microgravity based on our present data. LBNP simulates cardiovascular effects of gravity and is a potential countermeasure to microgravity. In this study we found that men are more responsive to LBNP compared to women. Treadmill exercise within LBNP showed less protective effect on bone resorption during bed rest in women as compared to men (Zwart et al. 2007).

### Tibial oxygenation response to lower body negative pressure

Previous studies document that muscle oxygenation in the calf decreases in response to LBNP (Hachiya et al. 2008). Similar to our previous study, LBNP did not significantly change tibial oxygenation in both men and women (Fig. 3) for all conditions. However the cerebral oxygenation is significantly higher in men compared to women. A previous study has shown that regional oxygenation saturation in the pre‐frontal cortex of men and women measured using the NIRS shows that deoxygenation levels are similar in men and women in contrast to our study (Smith and Billaut 2012). The tibial oxygenation levels are similar in men and women (Fig. 3). Leg oxygenation measurements using the NIRS record that men and women exhibit a similar arterial desaturation and are hence likely to have similar oxygen delivery rates in the tibia during LBNP (Smith and Billaut 2012). One suggestion is that the local fat differences may contribute to the differences observed since the NIRS signal interferes with fatty tissue. Women exhibit a greater fatty acid and carbohydrate metabolism than their male counterparts (Tarnopolsky 2000). Thus, the differential cerebral oxygenation rates between men and women may be related to tissue composition or fluid shifts. However, the latter has to be confirmed with additional experiments.

### Leg volume changes with lower body negative pressure

The calf volume includes both the capacitance response and total capillary filtration. Previous study demonstrates that calf volume increase was equivalent in both the genders during LBNP. (Lindenberger and Lanne 2007). As previously noted, the calf circumference increases in the first 3 min during LBNP, followed by slower and continuous rise caused by net trans capillary fluid filtration from blood to tissue (Olsen and Lanne 1998). The venous compartment in the legs have a larger impact of LBNP compared to pelvic or the abdominal regions (Lindenberger and Lanne 2007). This in turn elicits an increased sympathetic response with a higher peripheral resistance and increase in heart rate. Women are more susceptible to orthostatic stress than men and hence it is hypothesized that women have greater venous compliance in the legs, predisposing them to orthostatic intolerance. Pelvic pooling of blood in women during LBNP which may also contribute to the gender differences in LBNP responses. Laser Doppler studies showed that the under normal conditions, basal femoral arterial inflow to lower leg is the same in men and women, although the diameter of vessels in men is 52% more than women. The mean velocity of blood flow in women is 44% higher than the mean velocity in men (Holland et al. 1998). Men respond with greater vasoconstriction than women similar to our findings that men are more responsive to LBNP compared to women (Fig. 4). The calf circumference changes are due to venous pooling in the legs and responses to LBNP and follows the Starling‐Landis principle.

### Limitations

PPG values are only relative measurements of the bone microvascular flow and not absolute numbers. The NIRS can readily report on the blood volume and oxygenation, however the NIRS is reported to be problematic in measuring real time oxygen consumption. The NIRS signal is also affected by the presence of subcutaneous adipose tissue thickness at the site of measurements. Thus the apparent cerebral oxygenation maybe caused by a difference in fat tissue thickness. This difference was minimized by placing the probe in the region closer to bone with minimal fat.

## Conclusions

We document for the first time gender specific differences in tibial microvascular flow and cerebral oxygenation levels during lower body negative pressure. The gender specific differences in response to countermeasures such as LBNP highlight the importance of formulating gender specific countermeasure strategies for microgravity. Women have less tolerance to upright posture or gravitational stress as compared to men due to reduced ability to maintain venous return and cardiac output. This can impact exercise capacity and development of exercise‐based countermeasures. Hence, delineation of the mechanisms involved is necessary for developing suitable gender specific countermeasures. The underlying mechanisms resulting in the differential response to LBNP is not yet fully elucidated and warrants further investigation.

## Conflict of Interest

No conflict of interest, financial or otherwise.
